# Diagnosing Median Arcuate Ligament Syndrome in a Patient With a History of Duodenal Lymphoma: A Case Report

**DOI:** 10.7759/cureus.79735

**Published:** 2025-02-27

**Authors:** Laura Miranda Burgos, Alphonsa Thomas, Jordy B Godinez

**Affiliations:** 1 Internal Medicine, Broward Health North, Deerfield Beach, USA

**Keywords:** chronic abdominal pain, duodenal lymphoma, median arcuate ligament, median arcuate ligament syndrome, vascular disorder

## Abstract

Median arcuate ligament syndrome (MALS) is a rare vascular disorder that leads to a constellation of nonspecific gastrointestinal symptoms. We present a case of a 60-year-old male with a history of duodenal lymphoma in remission, insulin-dependent diabetes mellitus, and longstanding gastrointestinal symptoms, who developed worsening postprandial epigastric pain over eight months. Despite extensive evaluations including esophagogastroduodenoscopy (EGD), gastric emptying studies, and a hepatobiliary iminodiacetic acid (HIDA) scan, no definitive cause was identified. A computed tomography (CT) angiogram revealed anterior superior indentation of the proximal celiac artery by the diaphragmatic crus and post-stenotic dilation, consistent with MALS. The patient underwent laparoscopic median arcuate ligament release, resulting in significant symptom relief. This case underscores the importance of considering MALS in patients with chronic abdominal pain, even those with complex medical histories.

## Introduction

Median arcuate ligament syndrome (MALS), also known as celiac artery compression syndrome, is a rare condition characterized by the compression of the celiac artery by the median arcuate ligament, a fibrous band of the diaphragm [1]. It typically presents with nonspecific gastrointestinal symptoms, including postprandial abdominal pain, weight loss, and nausea, mimicking other gastrointestinal and vascular disorders [2,3]. Diagnosis requires high clinical suspicion and confirmatory imaging, such as computed tomography (CT) angiography, magnetic resonance (MR) angiography, or Doppler ultrasonography [4,5]. This case highlights the presentation, diagnostic workup, and management of MALS, emphasizing the importance of considering this diagnosis in patients with a complex medical history and chronic abdominal pain in order to improve their quality of life.

## Case presentation

**Figure 1:**
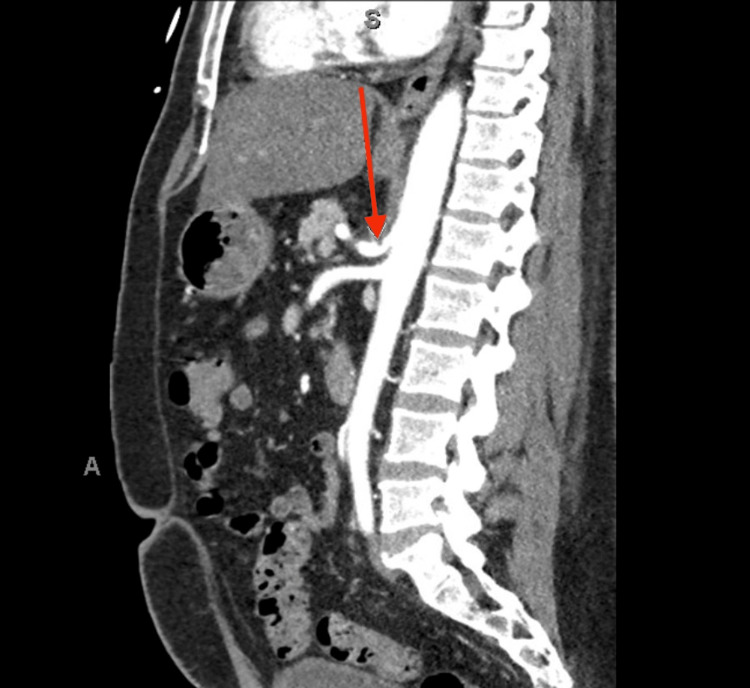
Computed tomography (CT) angiogram findings The CT angiogram revealed anterior superior indentation of the proximal celiac artery by the diaphragmatic crus with a J-shaped configuration and post-stenotic dilation (red arrow), consistent with MALS. MALS: median arcuate ligament syndrome

A 60-year-old male with a history of duodenal lymphoma in remission, insulin-dependent diabetes mellitus, and longstanding gastrointestinal symptoms presented with an eight-month history of worsening postprandial, dull-like, epigastric pain associated with nausea and intermittent vomiting that provided relief. He reported chronic watery diarrhea for over five years, more frequent when not using narcotic pain medications. He denied dysphagia, regurgitation of undigested food, or hematemesis. Physical examination revealed mild epigastric tenderness without peritoneal signs. Laboratory testing was non-contributory. Esophagogastroduodenoscopy (EGD) showed no active gastroduodenal pathology. Gastric emptying studies ruled out gastroparesis and a hepatobiliary iminodiacetic acid (HIDA) scan with cholecystokinin (CCK) demonstrated a normal gallbladder ejection fraction of 93%, excluding biliary dyskinesia. A computed tomography (CT) angiogram revealed anterior superior indentation of the proximal celiac artery by the diaphragmatic crus and post-stenotic dilation, consistent with MALS (Figure 1). No other significant abnormalities were noted.

The patient was referred to general surgery and scheduled for laparoscopic-assisted median arcuate ligament release, which ultimately provided symptomatic relief.

## Discussion

MALS is a rare condition caused by extrinsic compression of the celiac artery, leading to ischemia and neural irritation of the celiac plexus [6]. Symptoms often include postprandial epigastric pain, nausea, and unintentional weight loss due to food aversion, mimicking functional gastrointestinal disorders [2,3]. Diagnosing MALS requires a high index of suspicion, especially in patients with chronic abdominal pain unresponsive to conventional treatments or in those with complex medical histories, such as this case involving a history of duodenal lymphoma. Computed tomography (CT) angiography and magnetic resonance (MR) angiography can reveal characteristic findings, such as narrowing of the celiac artery with post-stenotic dilation, often more pronounced during expiration [4,5]. Additionally, Doppler ultrasonography can assess blood flow and detect compression of the celiac artery. However, these imaging modalities may not always be definitive; thus, clinical evaluation and imaging are often necessary for diagnosis [7].

Primary duodenal lymphomas are extremely rare, with a total of 1060 cases identified between 1998 and 2015 [8]. They can lead to chronic gastrointestinal symptoms, including pain, diarrhea, and malabsorption, which may persist even after remission due to treatment-related complications such as post-inflammatory changes, fibrosis, or altered gut motility [9,10]. Additionally, lymphoma-related mesenteric or retroperitoneal lymphadenopathy can contribute to vascular compression syndromes, further complicating the diagnostic picture [11]. In our patient, the longstanding gastrointestinal symptoms, including unintentional weight loss and episodic pain, initially raised concerns for either a recurrence of lymphoma or post-treatment complications, leading to an extensive workup. However, the absence of active disease on endoscopy and imaging, coupled with the characteristic findings on CT angiography, confirmed MALS as the underlying cause of his symptoms.

Management of MALS involves surgical decompression through the laparoscopic or surgical release of the median arcuate ligament, aiming to relieve vascular and neural compression of the celiac artery [3,12]. Postoperative outcomes are generally favorable, with symptom relief reported in most cases [13,14]. In patients with complex medical histories, such as a history of duodenal lymphoma in remission, the presentation of MALS can be particularly challenging. The overlap of symptoms between MALS and other gastrointestinal disorders necessitates a comprehensive evaluation to avoid misdiagnosis [15]. Additionally, the presence of comorbid conditions may influence the choice of a surgical approach and postoperative management. For instance, patients with a history of malignancy may have altered anatomy or an increased risk of complications, which should be considered when planning surgical intervention [16]. Early recognition and intervention are essential to reduce morbidity and improve quality of life [14].

## Conclusions

This case highlights the importance of considering MALS as a differential diagnosis in patients with chronic, unexplained gastrointestinal symptoms, particularly when conventional evaluations fail to identify a clear cause. Given its rarity and overlapping presentation with other gastrointestinal disorders, MALS requires a high index of suspicion and a thorough diagnostic workup, including CT angiography, to confirm the diagnosis. In patients with complex medical histories, such as prior malignancy, careful assessment is crucial to avoid misdiagnosis and unnecessary delays in treatment. Laparoscopic median arcuate ligament release remains the mainstay of therapy, offering significant symptomatic relief and improved quality of life. Early recognition and intervention are essential in optimizing outcomes for patients with this rare vascular disorder.
